# New Developments in the Pathogenesis, Therapeutic Targeting, and Treatment of H3K27M-Mutant Diffuse Midline Glioma

**DOI:** 10.3390/cancers13215280

**Published:** 2021-10-21

**Authors:** Davis P. Argersinger, Sarah R. Rivas, Ashish H. Shah, Sadhana Jackson, John D. Heiss

**Affiliations:** Surgical Neurology Branch, National Institute of Neurological Disorders and Stroke, National Institutes of Health, Bethesda, MD 20892, USA; davisarg@med.umich.edu (D.P.A.); sarah.rivas@nih.gov (S.R.R.); ashish.shah2@nih.gov (A.H.S.); sadhana.jackson@nih.gov (S.J.)

**Keywords:** biopsy, diffuse midline glioma, H3K27M-mutant, immunotherapy, diffuse intrinsic pontine glioma (DIPG), radiotherapy, chemotherapy

## Abstract

**Simple Summary:**

H3K27M-mutant diffuse midline glioma is a rare childhood cancer originating in midline brain structures. The H3K27M mutation substitutes an amino acid on histone H3 that promotes gene expression and tumor growth. This cancer has a dismal prognosis and requires new and better treatment approaches. This review discusses controversies regarding tumor biopsy and summarizes molecular tumor characteristics that are therapeutic targets. We describe preclinical studies and clinical trials utilizing immunotherapy, radiation, and chemotherapy against this cancer.

**Abstract:**

H3K27M-mutant diffuse midline gliomas (DMGs) are rare childhood central nervous system tumors that carry a dismal prognosis. Thus, innovative treatment approaches are greatly needed to improve clinical outcomes for these patients. Here, we discuss current trends in research of H3K27M-mutant diffuse midline glioma. This review highlights new developments of molecular pathophysiology for these tumors, as they relate to epigenetics and therapeutic targeting. We focus our discussion on combinatorial therapies addressing the inherent complexity of treating H3K27M-mutant diffuse midline gliomas and incorporating recent advances in immunotherapy, molecular biology, genetics, radiation, and stereotaxic surgical diagnostics.

## 1. Introduction

Diffuse intrinsic pontine glioma (DIPG) was recently reclassified as *diffuse midline glioma, H3K27M-mutant* in the 2016 World Health Organization (WHO) classification of central nervous system tumors [1,2]. H3K27M-mutant diffuse midline glioma (DMG) is the second most common childhood malignant brain tumor with an incidence of 200–300 cases annually in the United Stated [3]. Accounting for nearly two-thirds of childhood brainstem tumors, H3K27 diffuse midline gliomas typically present in 3–10 year old children, with a median overall survival of 9–12 months post diagnosis [4].

**Figure 1:**
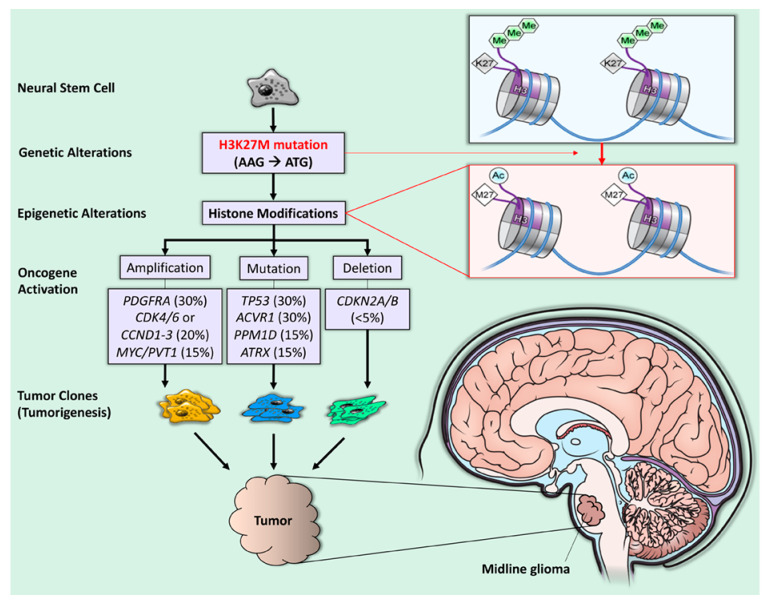
H3K27M mutation and tumorigenesis in diffuse midline glioma. The H3K27M mutation is a recurrent somatic gain-of-function missense mutation (AAG → ATG), resulting in a lysine 27 to methionine (p. Lys27Met: K27M) substitution in histone 3 (H3) variants (purple quadrant). The blue line represents double-stranded DNA wrapped around histones (short, segmented cylinders) regulating normal gene expression. The H3K27M mutation leads to the global loss of H3K27 trimethylation (green hexagons) and subsequent gain of H3K27 acetylation (blue circles), which is linked to oncogenesis (gene amplification, mutation, and deletion) and, subsequently, tumorigenesis [1].

“H3K27M” is the abbreviated descriptor of a recurrent somatic gain-of-function mutation, resulting from a lysine 27 to methionine (p.Lys27Met: K27M) substitution in histone 3 (H3) variant (Figure 1) [5,6]. The H3K27M mutation leads to the global loss of H3K27 trimethylation and subsequent gain of H3K27 acetylation, which has been linked to oncogenesis through upregulation of proto-oncogenes and suppression of cellular differentiation [7]. Despite only a single gain-of-function mutation, diffuse midline gliomas with the H3K27M mutation carry a worse prognosis than wild-type midline gliomas [1,8,9]. To better understand the reasons for the poor prognosis for H3K27 tumors, we reviewed the pathophysiology of these tumors and novel treatment strategies. This review highlights new developments in H3K27M diffuse midline glioma characterization related to epigenetic and targeted molecular therapy and therapeutic approaches that may prolong survival for these tumors. 

## 2. Clinical Overview

### 2.1. Characteristics and Presentation

H3K27M-mutant diffuse midline gliomas typically arise in the pons, infiltrate and expand throughout the brainstem, and cause progressive symptoms. Consequently, symptomatology of these tumors depends on the size and extent of infiltration. Nevertheless, patients typically present with hydrocephalus, pseudo-bulbar symptoms (dysphagia, slurred speech, and long-tract signs), lower cranial neuropathies, and headaches [10].

### 2.2. Diagnosis

**Figure 2:**
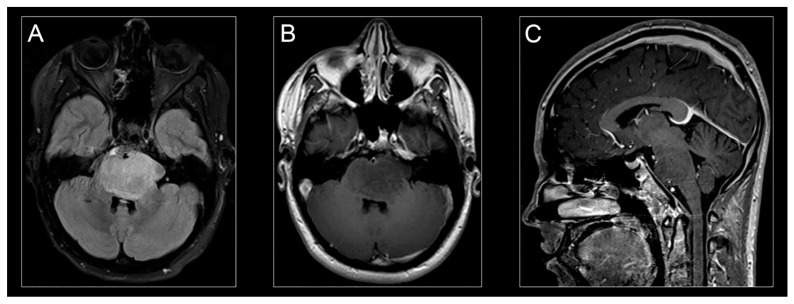
H3K27M-mutant diffuse midline glioma on MR imaging. Classic appearance of H3K27M-mutant diffuse midline glioma on MR-imaging: hyperintense signal on an axial T2-weighted fluid-attenuated inversion recovery (FLAIR) image (**A**), hypointense signal on an axial T1-weighted post-contrast image (**B**), and isointense signal on a sagittal T1-weighted post-contrast image (**C**).

Characteristic clinical symptoms and pathognomonic radiographic findings lead to a presumptive H3K27M-mutant diffuse midline glioma diagnosis. Conventional magnetic resonance imaging (MRI) typically demonstrates a hyperintense signal on T2-weighted images and expansion of the pons (taking up at least 2/3 of the pons) and sometimes adjacent brainstem. Enhancement patterns for H3K27-mutant diffuse midline gliomas vary and may not always be present at disease presentation (Figure 2A,C) [11].

Histological and molecular tumor characteristics traditionally have not guided treatment strategies in H3K27M-mutant DMG [12]. However, previous studies have shown that stereotactic biopsy of pontine tumors has relatively low morbidity and modest prognostic importance [13,14,15,16,17,18,19]. Thus, the development of molecularly guided treatments predicated on tumor biopsy tissue findings would enhance the diagnostic value of stereotactic biopsy [19].

Since repeat biopsy is rare for DMG patients, recent advances have suggested that liquid biopsies (through serum) could measure treatment response by detecting H3K27M mutations in circulating tumor DNA [20,21]. Specifically, levels of H3K27M plasma circulating tumor DNA may correlate with radiographic tumor responses and could be monitored before and after treatment. Table 1 demonstrates the accuracy of surgical biopsies in patients with H3K27M-mutant diffuse midline gliomas (94.5%) to obtain diagnostic tumor tissue and evaluate targetable molecular pathways [13,15,21,22,23,24,25].

### 2.3. World Health Organization Classification and Grade

The 2016 WHO classification of central nervous system tumors considers H3K27M-mutant diffuse midline glioma a high-grade lesion (WHO grade IV) [1]. Previous pathologic grading of DIPG depended on histologic appearance. Most tumors had a high-grade appearance, such as glioblastoma (WHO grade IV) or anaplastic astrocytoma (WHO grade III). However, some had lower-grade histology, such as a well-differentiated astrocytoma (WHO grade II) [1,26,27]. The presence of H3K27M mutation in 2016 raised the level of tumor malignancy to Grade IV even in cases having a low-grade histologic appearance. [28] Overall, detection of the H3K27 mutation serves as a more accurate prognosticator than conventional histopathology. As a result, neuropathologists have adopted a molecular biology-based classification system for the diagnosis of these tumors.

## 3. Pathogenesis: Stem Cell and Mutation

### 3.1. The Cell of Origin, H3K27M Mutation, and ACVR1 Mutation

Our current understanding of the molecular biology of H3K27M-mutant DMG has resulted from the analysis of tumor tissue obtained through stereotactic surgical biopsy or postmortem tissue procurement [5]. In a recent study of tumor tissue from 215 pediatric patients with H3K27M-mutant diffuse midline glioma, Castel et al. showed that the tumor gene expression profile was more closely tied to molecular tumor characteristics than tumor location or survival [29]. They also found that embryological differentiation (midline vs. hemispheric) more accurately characterized these tumor cells’ origin than the structure involved (e.g., brainstem vs. thalamus). To further characterize the H3K27M-mutant DMG cell of origin, Monje et al. identified cells of origin that resemble oligodendrocyte precursor cells. Their finding was supported by more recent RNA-seq experiments by Filbin et al. [30,31]. These findings support (1) the rationale in the updated 2016 WHO classification identifying DIPG more precisely by its genotypic and molecular profile (H3K27M-mutant), and (2) treating these tumors with molecularly driven rather than location-driven therapies. 

A recurrent somatic gain-of-function mutation, leading to a lysine 27 to methionine (p.Lys27Met: K27M) substitution in histone 3 (H3) variants, characterizes more than 85% of DMGs [5,6,32]. The K27M substitution affects histone variant H3.3 and H3.1, resulting from mutations to the *H3F3A* and *HIST1H3B/C* genes, respectively, with over 70% occurring in *H3F3A* [33]. The H3.3K27M gliomas are typically associated with loss-of-function mutations (p53) and gain of function of platelet-derived growth factor alpha (PDGFRA), while H3.1K27M gliomas have been associated with mutations in Activin A receptor type 1 (ACVR1). H3K27M mutation globally reduces H3K27 trimethylation (H3K27me3), leading to elevated expression of gliomagenesis genes [7,34,35]. Thus, research focused on reversing the H3K27 mutation effects through targeted upstream target inhibition or epigenetic therapy remains a viable treatment option [36,37,38].

Up to 32% of H3K27M-mutant diffuse midline glioma cases have *ACVR1* mutations. While these mutations are associated with gliomagenesis, previous studies have shown they are insufficient for inducing gliomagenesis alone [39,40]. Somatic *ACVR1* mutations are nearly exclusively limited to H3K27M-mutant diffuse midline glioma, occurring on only 0.3% of all other tumor types. These mutations induce hyperactive bone morphogenic protein (BMP) signaling, which arrests oligodendrocyte differentiation and increases tumorigenicity [36,37]. Specifically, Hoeman et al. found that mouse tumor models with *ACVR1* R206H plus H3.1K27M mutation had significantly shorter survival than tumor models with H3.1K27M mutations and no *ACVR1* mutation [39]. These findings suggest tumor-selective molecular therapy targeting ACVR1 mutations may decrease tumor aggressiveness and improve patients’ H3K27 mutant DMG life expectancy [37,41]. 

### 3.2. Therapeutic Targeting: Preclinical Development

The change in the 2016 WHO classification of DIPG to H3K27M-mutant diffuse midline glioma highlighted the need to identify potential therapeutic targets in the histologic and molecular tumor microenvironments. Furthermore, recognizing the roles of the histone mutation as the initiating feature and epigenetic modifiers as drivers of tumor pathogenesis has also influenced developing treatment strategies for treating H3K27M-mutant diffuse midline gliomas [1,40].

### 3.3. Targeting H3K27M Mutation

H3K27M-mutant DMG has a distinct epigenetic landscape characterized by dysregulated histone acetylation and methylation. One therapeutic option for these tumors would be histone-modifying drugs that reverse epigenetic silencing [42]. In a recent study, panobinostat, a histone deacetylase inhibitor, potently inhibited cell proliferation, viability, and clonogenicity of human and murine H3K27M cells in vitro [43]. In genetically engineered tumor-bearing mice, systemic panobinostat administration produced higher drug concentration in brainstem tumor tissue than normal brain tissue, reduced tumor cell proliferation, and increased H3 acetylation level [43]. In another preclinical study, panobinostat increased tumor cell trimethylation and acetylation and had an antitumor effect against H3K27M in vivo and in vitro [18]. Several recent studies have also investigated the role of chromatin remodeling in H3K27M-mutant DMG treatment. Ehteda et al. recent reported the efficacy of panobinostat and CBL0137, a compound that facilitates chromatin transcription (FACT), in prolonging the survival of mice bearing H3K27M orthografts [44]. Anastas et al. demonstrated that Corin, a bifunctional histone deacetylase and lysine-specific demethylase 1 inhibitor, increased H3K27Mme3 levels, which subsequently induces cell death, cell-cycle arrest, and cellular differentiation. The transcriptional changes associated with these inhibitors also corresponded to improved outcomes in DIPG patients [45], Relatedly, GSKJ4, an H3K27M demethylase inhibitor that increases H3K27me3 in H3K27M-expressing cells (transgenic cells with low H3K27me2/3 expression), had a potent, synergistic antitumor effect when paired with panobinostat [46]. Additionally, H3K27M mutation knockdown in a pontine glioma xenograft restored K27M-dependent loss of H3K27me3 and delayed tumor growth [47]. Collectively, these preclinical studies show that histone deacetylase and demethylase inhibitors counter H3K27M mutation effects in DMG models and are promising therapeutic agents, alone or in combination with other epigenetic drugs. 

### 3.4. Targeting ACVR1 Mutation

Although most preclinical studies of diffuse midline glioma target H3K27M-related mechanisms, *ACVR1* mutations remain an important driver of tumorigenesis in more than 30% of H3K27M-mutant diffuse midline gliomas [40,48]. Since ACVR1 encodes the serine/threonine kinase (ALK2), ALK2 inhibitors could play a role in H3K27M DMG treatment. Recently, ALK2 inhibition improved survival in orthotopic xenograft mice bearing H3.3K27M, ACVR1R206H tumors compared to control mice without the ACVR mutation [49]. A recent study investigated the clonal evolution of H3K27M-mutant DMG and demonstrated *ACVR1* mutations alongside H3K27M mutations in the earliest tumor clone, suggesting that *ACVR1* mutation is critical for oncogenesis [50]. Novel agents to target the mutated *ACVR1* receptor or its downstream effects are in development. A recent study identified that E6201 bound to wild-type and mutated ACVR1 inhibited activation of the ACVR1-encoded receptor, BMP1, and improved survival in mice harboring *ACVR1* and H3K27M mutations [41]. Therefore, future clinical trials of MEK1/2 inhibitors may show their effect on H3K27M-mutant diffuse midline glioma [51]. Additionally, the synergistic mechanism of *ACVR1* and H3K27M mutations in H3K27M-mutant diffuse midline gliomagenesis requires further study.

### 3.5. EZH2 Inhibition

Overexpression of enhancer of zeste homolog 2 (EZH2), an enzyme involved in histone methylation, has been associated with a more dismal prognosis in DMG patients with H3K27M mutations [52]. Mohammad et al. found that small-molecule EZH2 inhibitors abolished tumor growth in an H3K27M-mutant diffuse midline glioma mouse model by inducing protein p16^INK4A^, a tumor-suppressing molecule [53]. Zhang et al. found that EZH2 inhibition, when paired with bromodomain and extra-terminal (BET) protein inhibition, suppressed H3K27M neural stem cells and significantly altered H3K27M-mutant tumor cell proliferation [54]. Although both studies suggest that suppression of EZH2 is a promising therapeutic strategy, additional studies that singularly target EZH2 and not multiple targets are needed to gauge better whether EZH2 suppression alone is a viable strategy to arrest tumor proliferation and disease progression. Additionally, since polycomb repressive complex 2 (PRC2) has recently been implicated in H3K27M gliomas, targeting EZH2, the main enzymatic subunit of PRC2, may also be an attractive option for PRC2-directed therapy [37].

### 3.6. Metabolic Inhibitors

Several other approaches for targeting H3K27M DMG have also been proposed that target cellular metabolic pathways. Recently, Khan et al. demonstrated that polyamine synthesis is upregulated in DIPG and, therefore, could be targeted through a synthetic lethality-based approach using a polyamine synthesis inhibitor, difluoromethylornithine (DFMO). Since DIPG cells preferentially escape DFMO inhibition through upregulation of the polyamine transporter SLC3A2, adding polyamine transport inhibitors (AMXT 1501) potentiated tumor-selective cytotoxicity in vitro and in orthotopic animal models [55]. Additionally, mitochondrial dysfunction has also been implicated in pediatric high-grade gliomas. Because tumor tissue has a marked reduction in mitochondrial DNA compared to normal controls, targeting this inherent deficiency by shifting glucose metabolism to mitochondrial-dependent oxidative phosphorylation has also been proposed. Recently, Shen et al. demonstrated the efficacy of this strategy by utilizing a combination of dichloroacetate (DCA), metformin, and radiation to induce cellular death by inducing mitochondrial dysfunction [56]. These strategies leverage the distinct metabolic landscape of pediatric diffuse midline gliomas and could be potentially translated into clinical studies.

### 3.7. Immunotherapy

Several studies have investigated immunotherapy as a potential therapy for H3K27M DMG. Preclinical research for H3K27M DMG has incorporated recent chimeric antigen receptor (CAR) T-cell therapy advances. CAR T-cell therapy directed against GD2 (disialoganglioside), a tumor-associated cell surface antigen, is intriguing because patient-derived H3K27M DMG cell lines and neuroectodermal tissues almost uniformly express GD2 [57]. Additionally, in vitro exposure to anti-GD2-CAR T cells significantly depleted cultured H3K27M-mutant cells in a dose-dependent manner [57]. However, the brain penetrance of this CAR T-cell directed therapy is uncertain. Mount et al. did demonstrate brain penetrance in their murine xenograft model, although such penetrance was associated with neurotoxicities including peritumoral inflammation and resultant hydrocephalus [57]. Nevertheless, the tumoricidal effect of GD2-targeted CAR T cells in in vitro assays supports further development and testing of this approach in animal models. Interestingly, immunotherapeutic trials for neuroblastoma, osteosarcoma, and melanoma which target GD2 were successful [57].

Immunotherapy using tumor-infiltrating lymphocytes (TILs) directed to the tumor microenvironment has been tested in other solid tumors [58]. However, the harvesting of TILs in DMGs is problematic because of the small volume of tumor tissue obtained by stereotactic biopsy. Compared to other solid tumors such as melanoma and breast cancer, there are relatively few TILs within a brainstem tumor biopsy from which enough TILs can be grown [58]. Scheper et al. proposed reactivation of the TCR repertoire on cancer cells to augment present CAR T cell and checkpoint-inhibition therapies. This strategy seeks to (1) increase the quality of T-cell receptors (TCRs) in the intratumoral microenvironment, (2) reactivate intratumoral T cells, (3) enhance TIL chemotaxis and TIL infiltration, and (4) increase anti-DMG immune effects [59]. In a recent study of the immune microenvironment of H3K27M-mutant DMG, Lieberman et al. demonstrated [60] minimal macrophage or T-cell infiltration compared to nontumor controls [61]. Additionally, transcriptomic studies using bulk and single-cell RNA-seq demonstrate a relatively bland immune landscape (low expression of cytokines, absent PDL-1) in the DIPG microenvironment. This nonactivated immune phenotype coupled with the absence of infiltrating lymphocytes may diminish the efficacy of H3K27M DMG immunotherapy. On the other hand, these findings suggest that enhancing T-cell recruitment, activation, and retention of tumor-specific effector immune cells within the tumor microenvironment could potentiate immunotherapy for H3K27M-mutant DMG [57,59,61].

## 4. Clinical Management of H3K27M

### 4.1. Challenges

H3K27M-mutant diffuse midline gliomas infiltrate and proliferate within eloquent brainstem structures and are not amenable to surgical resection. Regrettably, standard therapies for H3K27M-mutant DMG remain palliative. Yet, a diagnostic biopsy helps confirm tissue diagnosis for both prognostication and potential enrollment in clinical trials. Biopsy tissue is also valuable for establishing tumor cell lines to test and validate novel treatments in cell culture or animal models. Nevertheless, significant efforts to improve the treatment of this devastating childhood tumor continue.

### 4.2. Radiation Therapy

Radiation therapy is the only treatment that improves life expectancy. A 54–60 Gy radiotherapy tumor dose over 6 weeks has been the standard treatment recommendation for H3K27M-mutant diffuse midline glioma over the past 20 years, delaying tumor progression for up to 3 months in 70–80% of patients [2,62,63]. Hyperfractionated therapy is less effective than conventional therapy and may increase radiation toxicity and other morbidities [64,65,66]. However, hypofractionated radiotherapy may reduce the burden of treatment for caretakers by significantly reducing hospitalizations and improving the quality of life [67,68]. 

Radiation may also have synergistic effects if combined with chemotherapy or immunotherapy for treating H3K27M-mutant DMG. Radiosensitizing agents such as gemcitabine selectively target rapidly dividing tumor cells and potentiate radiotherapy. In a phase I/II study, gemcitabine, a pyrimidine analog, and concurrent radiotherapy were well tolerated and without dose-limiting toxicity. However, overall survival was similar to that of historical controls (mOS of 8.7 months) [69]. Another trial for DIPG used the oral nucleoside analog, capecitabine, concomitantly with radiotherapy and demonstrated worse progression-free survival (PFS) at 1 year than historical controls (7.2% vs. 15.6%, *p* = 0.007) and no improvement in median overall survival (Table 2) [70]. In a phase III multicenter study, Fleischhack et al. combined standard radiotherapy with nimotuzumab, a recombinant humanized monoclonal antibody against the human epidermal growth factor receptor (EGFR) [71]. They found that nimotuzumab administered concurrently with standard 54 Gy external beam radiotherapy over 6 weeks was as efficacious and well-tolerated as a chemotherapy and radiotherapy combination. Combination radiotherapy and nimotuzumab provided patients with a treatment regimen that permitted shorter outpatient clinic visits and longer intervals between treatments than chemotherapy alone [71]. While additional studies are needed to identify optimal patient populations of DMG that would benefit from combining nimotuzumab with radiotherapy, reducing hospitalizations would improve the overall quality of life for these patients with such poor survival. 

Kline et al. recently reported a study of the efficacy of combination re-irradiation and PD-1 inhibition using nivolumab in 31 patients with H3K27M-mutant diffuse midline glioma [74]. Patients who received re-irradiation therapy had prolonged overall survival compared to those who were not re-irradiated; combining re-irradiation therapy with nivolumab slightly prolonged overall survival from both diagnosis and re-irradiation administration compared to re-irradiation alone. Compared to standard treatment, combined re-irradiation and PD-1 inhibition extended overall survival by more than 20% after diagnosis, but not more than re-irradiation alone [4,74].

### 4.3. Tumor-Localized Therapy 

Technological and neurosurgical advancements in stereotactic MRI-guided infusion catheter placement, catheter design, and drug distribution monitoring have paved the way for clinical studies and trials of intratumoral drug delivery via convection-enhanced delivery (CED) [75]. CED is a regional drug delivery method utilizing small hydrostatic pressure gradients to drive the bulk flow of a drug through the extracellular spaces of the central nervous system. CED is a promising therapeutic delivery technique for treating patients with H3K27M-mutant diffuse midline glioma. Several earlier studies have established the technical parameters for safely using CED in patients with H3K27M-mutant DMG [76,77,78,79].

Two clinical trials using CED to treat H3K27M-mutant diffuse midline glioma have recently been reported. Heiss et al. investigated the safety, infusion distribution, and potential efficacy of CED of IL13–*Pseudomonas* exotoxin in five pediatric patients with H3K27M-mutant diffuse midline glioma [10]. They observed short-term radiographic antitumor effects in two of the five patients treated. However, the IL13–*Pseudomonas* exotoxin was not distributed widely enough to reach the entire MRI-defined tumor volume in any patient. Poor target drug distribution contributed to the lack of efficacy in this trial. Drug distribution could have been improved by infusing through multiple catheters or priming the tumor microenvironment. In another clinical trial, Souweidane et al. used CED to safely infuse a murine monoclonal antibody targeting glioma-associated B7-H3 antigen, conjugated to the radioisotope conjugate ([^124^I]-8H9) into the brainstem of pediatric patients with H3K27M-mutant DMG [72]. The primary endpoint was the maximum tolerated dose of [^124^I]-8H9 administered by convection-enhanced delivery. No dose-limiting toxicities occurred with any dose, and the maximum-tolerated dose was not reached. Overall survival in this group was slightly better than historical controls (15.3 months). Survival increased more in patients in higher-dose cohorts. 

These studies confirm that CED can be used to deliver therapeutic agents safely and effectively to the brainstem in high intratumoral doses without systemic exposure or toxicity. Several other clinical trials are building upon the studies mentioned previously [10,72,75]. An ongoing clinical trial (NCT01502917) continues to investigate the intratumoral binding ability and tumor growth suppression of [^124^I]-8H9 delivered by CED to the brainstem tumor with repeated CED infusions in pediatric patients [80]. Another clinical trial is investigating the safety and efficacy of CED-infused panobinostat for H3K27M-mutant tumor suppression (NCT03566199). Panobinostat, discussed previously regarding its ability in preclinical studies to counter the effects of the H3K27M mutation, has minimal penetrance of the blood–brain barrier (BBB) [43,81,82]. To circumvent the BBB, this trial uses CED to co-infuse nanoparticle-derived panobinostat (MTX110) and gadoteridol and MRI to track infusate delivery in near real-time. Lastly, a phase I study of CED of nanoliposomal irinotecan and gadolinium is ongoing for pediatric patients with newly diagnosed H3K27M-mutant diffuse midline glioma who have already completed focal radiotherapy (NCT03086616). Nanoliposomal irinotecan is a novel and highly stabilized irinotecan formulation that has shown promise for glioma treatment in vitro and in preclinical studies [83]. 

Tumor-localized therapies that do not require tumor catheters have also been proposed for H3K27M DMG. Super-selective intraarterial cerebral infusion is a novel tumor-localized therapeutic platform. This technique typically requires a primer such as mannitol to temporarily open the blood–brain barrier during arterial infusion. Recently, 10 treatment-refractory DMG patients received a super-selective intraarterial cerebral infusion of bevacizumab and cetuximab with minimal toxicity. Overall survival in this Phase 1 trial (NCT01884740) was 17.3 months from diagnosis, and several patients had radiographic responses [73]. Magnetic resonance-guided focused ultrasound (MRgFUS) is another recently developed method of delivering tumor-localized therapy. MRgFUS uses tumor-targeted ultrasonic waves with microbubbles to temporarily increase vessel permeability and open the BBB [84]. FUS increased intratumoral drug concentration up to fivefold in murine models with minimal toxicity. In orthotopic murine models of DMG, MRgFUS improved drug delivery to the tumor and decreased tumor growth [84,85,86,87]. Overall, these trials suggest that tumor-localized treatment may be an attractive option for H3K27M DMG and synergize with conventional chemotherapy and radiation regimens.

### 4.4. Immunotherapy 

Immunotherapy of H3K27M-mutant diffuse midline glioma is being tested as an adjuvant to traditional chemoradiation. Immunotherapy was safe when administered intravenously and concomitantly with radiotherapy [71,72,74]. Benitez-Ribas et al. recently reported a phase Ib immunotherapy clinical trial using autologous dendritic cells pulsed with an allogeneic tumor cell-line lysate to reactivate tumor-specific T cells in patients with newly diagnosed H3K27M-mutant diffuse midline glioma after irradiation [88]. Autologous dendritic cell vaccines were feasible, safely prepared, and generated an H3K27M-mutant diffuse midline glioma-specific immune response detected in peripheral blood mononuclear cells (PBMC) and cerebrospinal fluid (CSF). In another study, Fried et al. demonstrated that the immune-modulating antibody MDV9300 (pidilizumab) is a potentially promising treatment for H3K27M-mutant DMG following radiotherapy [89]. Of the nine pediatric patients enrolled in the study, two were still alive nearly 30 months from diagnosis at the trial’s conclusion, with radiographically defined disease stability [4,89]. Additionally, Tejada et al. reported the novel intratumoral use of DNX-2401, a replication-competent, genetically modified virus that stimulates an antitumor immune response, in a pediatric patient with H3K27M-mutant diffuse midline glioma [22]. Preclinical studies suggested that DNX-2401 (known as Delta-24-RGD) effectively induced both an oncolytic and an antitumor immune response in pediatric high-grade gliomas [90]. DNX-2401 is under investigation in an ongoing phase I clinical trial (NCT03178032) for recently diagnosed H3K27M-mutant DMG patients. Their findings further suggest that immunotherapy is a promising adjunctive treatment for H3K27M-mutant diffuse midline glioma. 

A review of recent studies and trials investigating various immunotherapeutic methodologies for the treatment of H3K27M-mutant diffuse midline glioma is shown in Table 3. Immunotherapy responses vary among patients. A better understanding of immune and tumor biomarkers may optimize and predict the effectiveness of immunotherapy in individual patients.

### 4.5. Quality of Life

Since H3K27M DMG treatment remains largely palliative at this time, the potential benefit of treatment should outweigh treatment-related side-effects affecting the patient’s quality of life (QOL). Treatment plans must be developed compassionately and empathically, bearing in mind the currently limited effectiveness of H3K27M-mutant diffuse midline glioma treatment. The median survival for H3K27M-mutant diffuse midline glioma is 9–12 months, and 2 and 5 year survival rates are 10% and 1%, respectively [2]. A recent phase I trial of convection-enhanced delivery of an IL13-targeted exotoxin for H3K27-mutant diffuse midline glioma used the 43-item Impact of Pediatric Illness (IPI) Parent Report Form to record health-related QOL in five pediatric patients [10]. Parent-reported QOL scores generally aligned with physical function and radiographic data. In two of five patients who showed a treatment response, QOL scores corresponded with improved Karnofsky Performance Status (KPS)/Lanksy. Performance Status (LPS) scores. Incorporating standard QOL measures into study designs would allow a more ready comparison of the effect of experimental treatments on the quality of life across studies.

### 4.6. Summary and Future Directions

H3K27M-mutated diffuse midline glioma is a devastating childhood central nervous system tumor with a dismal prognosis. Consequently, there is a desperate need for novel and effective therapeutic strategies to improve care for affected children. Recent advances in understanding the molecular biology of H3K27M-mutant diffuse midline glioma have driven research efforts. Ongoing laboratory studies and clinical trials aim to develop better chemotherapy, radiotherapy, immunotherapy, and intratumoral targeted treatments for H3K27M-mutated diffuse midline glioma. Combination therapies hold great promise for improving the present poor prognosis for H3K27M-mutant DMG patients. Basic and translational research findings provide insights into pathology, disease mechanisms, and novel therapeutic strategies for clinical trials. An improved understanding of the effects of tumor genetics, epigenetics, along with the immune microenvironments linked to patient outcomes, may lead to treatments better tailored to these factors. Therapeutic agents must reach the tumor to be effective. Thus, improved delivery techniques and distribution of therapeutics to the tumor are vital to enhancing the treatment of this presently incurable disease.

## 5. Conclusions

H3K27M-mutated diffuse midline glioma affects young children and has a dismal prognosis. Over the past 10 years, the name of this tumor was changed from DIPG to H3K27M-mutated diffuse midline glioma after its mutation was identified and its prognosis was found to relate better to the tumor’s mutation than to its histological appearance. Identification of molecular pathways proceeding from the H3K27M-mutatation to tumorigenesis has uncovered potential therapeutic targets. In vitro and animal models for H3K27M-mutated diffuse midline glioma provide the opportunity to perform preclinical studies with novel therapies. Clinical trials have showed that therapeutic platforms, such as convection enhanced delivery, blood-brain barrier opening, and immunotherapy, were tolerated well by patients with H3K27M-mutated diffuse midline glioma. Although clinical trials have not yet increased life expectancy, they paved the way for future trials using novel agents that showed efficacy in preclinical models. Patients with H3K27M-mutated diffuse midline glioma direly need better treatment. Scientists and clinicians are learning more about the molecular pathways driving tumor growth and invasion and are working tirelessly to develop more effective therapies for this devastating condition that improve the lives of affected patients. 

## Figures and Tables

**Table 1:** Surgical biopsy for patients with H3K27M-mutant diffuse midline glioma. Recent clinical studies and trials utilizing surgical biopsy for patients with H3K27M-mutant diffuse midline glioma.

Pathologic Diagnosis—Biopsied Tissue							
Technique and Trajectory	Reference	Patients (*N*)	Confirmed DIPG/DMG (*N*)	Other Diagnosis (*N*)	Non-Diagnostic (*N*)	Complications ^#^ (*N*)	No Complications ^#^ (*N*)
SSB (15), RASB (1): TF (5); TC (11); Open (5)	Pfaff et al. (2019) [15]	21	18	3	0	2	19
SSB; TC	Mueller et al. (2019) [21]	15	11	4	0	0	15
RASB; TC (9); TF (2)	Dawes et al. (2018) [13]	11	7	3	1	0	11
SSB; TC	Tejada et al. (2018) [22]	1	1	0	0	0	1
SSB; trajectory not reported	Plessier et al. (2017) [23]	18	18	0	0	(not reported)	(not reported)
RASB; TF	Carai et al. (2017) [24]	7	6	1	0	1	6
SSB; TC	Puget et al. (2015) [25]	130	130	0	0	5	125
SSB; TC	Gupta et al. (2018) [19]	50	48	0	2	1	49
-	Total:	253	239/253 (94.5%)	11/253 (4.3%)	3/253(1.2%)	9/253 (3.6%)	244/253 (96.4%)

^#^ Complications reported as severe, unresolved, and attributable to the surgical procedure; RASB: robot-assisted stereotactic biopsy; SSB: standard stereotactic biopsy; TC: transcerebellar; TF: transfrontal.

**Table 2:** Drug delivery for H3K27M-mutant diffuse midline glioma. Recent drug delivery studies and trials for the treatment of H3K27M-mutant diffuse midline glioma.

Treatment Delivery Method	Reference	Patients (*N*)	Drug	Outcome	Comments
Biopsy Tract	Tejada et al. (2018) [22]	1	DNX-2401	Drug was safely infused and well tolerated; no infusion-related or viral toxicity reported	Phase 1 trial ongoing (NCT03178032)
CED	Heiss et al. (2019) [10]	5	IL13–*Pseudomonas* exotoxin	MOS: 16.8 months MSTAD: 4.8 months Vd/Vi: 1:6/1 to 4.1:1	Short-term radiographic antitumor effects in 2/5 patients
	Souweidane et al. (2018) [72]	28	[^124^I]-8H9	MOS: 15.3 months Vd/Vi: 3.4:1	Average lesion-to-whole-body ratio of absorbed radiation dose higher than 1200
IV	Veldhuijzen van Zanten et al. (2017) [69]	9	Gemcitabine	PFS: 4.8 months MOS: 8.7 months QoL improved during treatment	Gemcitabine was safe and well tolerated with concomitant radiotherapy
Oral	Kilburn et al. (2018) [70]	44	Capecitabine	Capecitabine-treated cohort: 1 year PFS in 7.21% of subjects Capecitabine did not improve the outcomes of children with newly diagnosed DIPG	Capecitabine was rapidly absorbed and converted to its metabolites
Intraarterial	McCrea et al. (2021) [73]	10	Cetuximab Bevacizumab	Toxicity: grade 1 adverse events (40%) MOS: 17.3 months	Radiographic responses noted; Stable disease (*n* = 5) on FLAIR

BBB: blood–brain barrier; CED: convection-enhanced delivery; IV: intravenous; MOS: median overall survival; MSTAD: median survival time after drug; PFS: progression-free survival; QoL: quality of life; Vd/Vi: volume of distribution/volume of infusion.

**Table 3:** Immunotherapy for H3K27M-mutant diffuse midline glioma. Recent immunotherapy studies and trials for the treatment of H3K27M-mutant diffuse midline glioma.

Therapeutic Target	Reference	(*N*)	Therapy	Outcome	Comments
GD2	Mount et al. (2018) [57]	(Murine xenograft model)	CAR T-cell therapy	Anti-GD2 Car T cells demonstrated robust antigen-dependent cytokine generation and killing of H3K27M-mutant diffuse midline glioma cells	Patient-derived H3K27M-mutant glioma cell cultures were used
Tumor lysate-specific T Cells	Benitez-Ribas et al. (2018) [88]	9	Cell-mediated using ADCV	Specific antitumor lysate cellular response was identified in 8/9 patients	ADCV administration was feasible and safe, and it generated a DIPG-specific immune response detected in PBMC and CSF
PD-1	Fried et al. (2018) [89]	9	Immune modulating antibody MDV9300 (pidilizumab)	MES: 9.3 months MOS: 15.6 months	2 children were still alive nearly 30 months after diagnosis, with stable disease
Rb pathway-deficient H3K27M-mutant cells	Tejada et al. (2018) [22]	1	Cell-mediated using DNX-2401	No infusion-related or viral toxicity reported	Phase 1 trial ongoing (NCT03178032)

ADCV: autologous dendritic cell vaccines; CSF: cerebrospinal fluid; GD2: disialoganglioside expressed on tumors of neuroectodermal origin; MES: median event-free survival; MOS: median overall survival; PBMC: peripheral blood mononuclear cells; PD-1: programmed cell death protein 1.
